# Minority Stress and the Effects on Emotion Processing in Transgender Men and Cisgender People: A Study Combining fMRI and ^1^H-MRS

**DOI:** 10.1093/ijnp/pyab090

**Published:** 2021-12-08

**Authors:** Meltem Kiyar, Mary-Ann Kubre, Sarah Collet, Sourav Bhaduri, Guy T’Sjoen, Antonio Guillamon, Sven C Mueller

**Affiliations:** Department of Experimental Clinical and Health Psychology, Ghent University, Belgium; Department of Experimental Clinical and Health Psychology, Ghent University, Belgium; Department of Endocrinology, Ghent University Hospital, Belgium; Department of Experimental Clinical and Health Psychology, Ghent University, Belgium; Department of Molecular and Clinical Cancer Medicine, University of Liverpool, Liverpool, United Kingdom; Department of Endocrinology, Ghent University Hospital, Belgium; Department of Psychobiology, National Distance Education University, Madrid, Spain; Department of Experimental Clinical and Health Psychology, Ghent University, Belgium

**Keywords:** Emotion, functional neuroimaging, minority stress, spectroscopy, transgender men

## Abstract

**Background:**

Minority stress via discrimination, stigmatization, and exposure to violence can lead to development of mood and anxiety disorders and underlying neurobiochemical changes. To date, the neural and neurochemical correlates of emotion processing in transgender people (and their interaction) are unknown.

**Methods:**

This study combined functional magnetic resonance imaging and magnetic resonance spectroscopy to uncover the effects of anxiety and perceived stress on the neural and neurochemical substrates, specifically choline, on emotion processing in transgender men. Thirty transgender men (TM), 30 cisgender men, and 35 cisgender women passively viewed angry, neutral, happy, and surprised faces in the functional magnetic resonance imaging scanner, underwent a magnetic resonance spectroscopy scan, and filled out mood- and anxiety-related questionnaires.

**Results:**

As predicted, choline levels modulated the relationship between anxiety and stress symptoms and the neural response to angry and surprised (but not happy faces) in the amygdala. This was the case only for TM but not cisgender comparisons. More generally, neural responses in the left amygdala, left middle frontal gyrus, and medial frontal gyrus to emotional faces in TM resembled that of cisgender women.

**Conclusions:**

These results provide first evidence, to our knowledge, of a critical interaction between levels of analysis and that choline may influence neural processing of emotion in individuals prone to minority stress.

**Significance Statement**

In this study of 30 transgender men, 30 cisgender men, and 35 cisgender women, we uncovered that the brain metabolite choline appeared to play a critical role in modulating anxiety and perceived stress rates in people with experience of minority stress (i.e., transgender people) and their neural response to negative and emotionally ambiguous stimuli in the amygdala. Importantly, this effect was not visible for positive emotions or in people not affected by minority stress (cisgender comparisons). Therefore, we strongly believe these data provide exciting new leads in how choline might be implicated in both anxiety (but not depressive symptoms) and in modulating neural responses during emotion processing.

## Introduction

Transgender (hereafter trans) people are individuals who identify with a gender different to their sex assigned at birth and who may experience stigmatization as well as discrimination and transphobia-related violence (Meyer 2003; Bradford et al., 2013; European Union Agency for Fundamental Rights, 2014; White Hughto, 2015). As a result of such minority stress, trans people report high rates of anxiety, depression, and suicidal ideation (Mustanski and Liu, 2013; Heylens et al., 2014; Marshall et al., 2016). However, the neurobiological and neurochemical correlates of this experience have not yet been determined.

Neuroimaging work has shown that increased sensitivity towards stress can lead to permanent changes in the brain such as increased neural activation of the amygdala (Koch et al., 2016), amygdala volume decrease (Hanson et al., 2015), or continuous secretion of cortisol (Roberts and Lopez-Duran, 2019), all of which may have a negative impact on an individual. For instance, people with anxiety disorders might overgeneralize and perceive more neutral stimuli as threatening (Newman et al., 2013; Lissek et al., 2014). The same might be true for individuals experiencing minority stress who are vigilant towards their environment and vulnerable towards negative experience (Meyer 2003). How exactly brain neurochemistry such as brain metabolites influence affective responding in individuals affected by minority stress remains unknown. In fact, little is known how the underlying neurochemistry may influence neural responding. One possibility to examine this influence is by combining functional magnetic resonance imaging (fMRI) and proton magnetic resonance spectroscopy (^1^H-MRS). ^1^H-MRS enables assessment of brain metabolites playing critical roles in brain functioning and include choline (Cho), N-acetyl-aspartate, glutamate, myo-inositol, glycine, and creatine (Tran et al., 2009; Ip et al., 2017).

One such combined study in volunteers (Stan et al., 2014) revealed that increases in glutamate concentration correlated positively with anterior cingulate cortex (ACC) activation while processing angry faces. Adding to these findings, a group of researchers (Wise et al., 2020) has shown that administering a low dose of Cho reduced activity in the ACC and the amygdala during presentation of fearful stimuli in people with anxiety symptoms. Behaviorally, the link between Cho and anxiety was further cemented in a large study (n = 5918) of plasma concentrations. Here, another research group (Bjelland et al., 2009) documented an inverse effect, that is, individuals with low Cho levels had higher anxiety symptoms. This finding was later replicated by other researchers (Howells et al., 2015) who reported in the putamen that lower relative Cho concentrations were associated with higher anxiety levels in people with social anxiety only. Yet, the same study (Howells et al., 2015) additionally found a reverse effect (higher relative Cho concentrations associated with higher anxiety symptoms) in the thalamus for both the social anxiety and comparison group, suggesting regional specificity of metabolite effects on neural responding. Of note, the research group that found an inverse effect (Bjelland et al., 2009) did not find any associations of Cho with depressive symptoms. The striking presence of Cho in these prior findings may be linked to the fact that this metabolite is essential to produce acetylcholine (ACh) for cholinergic neurotransmission, where ACh is a neurotransmitter that plays a pivotal role in regulating mood and cognitive functions (Giacobini and Pepeu, 2018). Besides, Cho is also involved in 2 other crucial processes. First, Cho ensures the structural integrity and lipid-derived signaling for cell membranes, and Cho supports the methylation of cells, which, among others, is crucial for regulating hormones and producing energy (Giacobini and Pepeu, 2018). Taken together, these findings suggest that metabolites, especially Cho, are implicated in people high in anxiety symptoms that in turn might affect neural processing of emotions. To date, the link between Cho levels and emotion regulation in trans people has not been examined yet.

To our knowledge, only 1 neuroimaging study so far has examined affective processing in trans people. Recently a study (Beking et al., 2020) documented that gender-affirming hormonal treatment (administration of testosterone) increased right amygdala lateralization in trans boys (n = 21, age approximately 16 years). However, there were no clear sex differences and no specific findings with regards to emotion processing. Yet, pertinent to the idea of perceiving neutral or ambiguous stimuli as negative in individuals exposed to minority stress due to hypervigilance (Meyer 2003), little is known about how emotionally ambiguous stimuli (e.g., surprise) would be processed in such a cohort. Unfortunately, findings in studies assessing ambiguous emotions in cisgender (hereafter cis) people are presently disjunct (Browning and Harmer, 2012; Vrticka et al., 2014; Davis et al., 2016). Whereas the first study (Browning and Harmer, 2012) documented an increase in the ACC but not the amygdala during presentation of surprised faces, the second study (Davis et al., 2016) reported an increase in amygdala activation during unpredictable vs predictable surprise faces, respectively. The latest study (Vrticka et al., 2014) found an increased amygdala activation towards both positive and negative surprise stimuli. While disparate, these findings point at the potential utility to assess ambiguous emotions in trans people.

Therefore, this study addressed an important gap: to examine the influence of brain metabolites on the amygdala during (ambiguous) emotion processing in trans men, cis men, and cis women. Due to (1) experienced minority stress and increased vigilance towards the environment in minority group individuals (Meyer and Dean, 1998; Meyer, 2003), (2) higher rates of anxiety in trans people (Heylens et al., 2014), and (3) prior knowledge of an involvement of Cho in anxiety (Bjelland et al., 2009; Howells et al., 2015; Wise et al., 2020) we hypothesized that (1) trans relative to cis individuals would show larger neural responses to both negative and ambiguous stimuli (i.e., angry and surprise faces), and (2) that this effect would be modulated by Cho levels.

## METHODS

### Participants

Ninety-five (30 trans men [TM], 30 cis men [CM], and 35 cis women [CW]) participants completed the fMRI task and amygdala ^1^H-MRS and filled out the questionnaires in exchange for monetary compensation (20 Euros). Ten participants were excluded after data collection because of technical/quality issues during task fMRI and ^1^H-MRS, resulting in no or low-quality data. Consequently, the fMRI sample consisted of 26 TM (M_age_ = 28.23, SD = 11.51), 29 CM (M_age_ = 29.79, SD = 6.76), and 30 CW (M_age_ = 30.6, SD = 8.97). For ^1^H-MRS, 2 additional participants had to be excluded after quality assessment. Hereby, the ^1^H-MRS sample consisted of 25 TM (M_age_ = 27.78, SD = 26.64), 28 CM (M_age_ = 26.64, SD = 6.83), and 30 CW (M_age_ = 30.83, SD = 9.04). All participants, who were ≥18 years old, were age matched but differed in education, resulting in higher levels of education for the cis group relative to TM (*P < *.001, $\eta_{p}^{2}=.423$). Therefore, all findings included education as a covariate of no interest. As expected, groups also differed on trait anxiety (*F*_(2,92) = _8.45, *P < *.001), state anxiety (*F*_(2,92) _=_ _4.51, *P = *.01), depression (*F*_(2,92) _=_ _6.78, *P < *.01), and perceived stress scores (*F*_(2,92) _=_ _3.25, *P = *.04) (cf. Table 1). Since the demographics with and without poor-quality fMRI/^1^H-MRS did not differ significantly, all data are shown in the table for sake of comprehensiveness. TM were recruited through the Department of Endocrinology and Center for Sexology and Gender of Ghent University Hospital, Belgium. Cis people were recruited through word of mouth and flyers. Exclusion criteria were neurological disorders and history of hormonal medication (i.e., only hormone-naïve TM prior to gender-affirming hormonal treatment were recruited). All participants provided written informed consent prior to the start of the study, and ethical approval was granted by the ethical committee of Ghent University Hospital, Belgium.

**Table 1.** Demographic information.

Variable	TM (n=30)	CM (n=30)	CW (n=35)	*P* value	Effect size ηp2
Age (mean/SD)	27.39 (10.87)	26.80 (6.64)	30.46 (9.60)	*ns*	.031
Ethnicity	White (Flemish/Dutch)				
Education^a^	—	—	—	<.001	.489^b^
Primary school	3 (10%)	0	0		
High school	21 (70%)	11 (37%)	12 (34%)		
Graduate school	6 (20%)	7 (23%)	12 (34%)		
Postgraduate	0	12 (40%)	11 (31.4%)		
Depression (BDI)	12.03 (8.56)	6.03 (4.94)	7.20 (6.26)	<.01_c_	.128
State anxiety (STAI)	41.57 (9.01)	35.97 (7.74)	36.31 (7.79)	.01_c_	.089
Trait anxiety (STAI)	43.73 (10.12)	34.13 (8.94)	34.63 (11.44)	<.001_c_	.155
Perceived stress (PSS)	15.77 (6.745)	12.00 (5.213)	13.94 (5.16)	.04_d_	.066
MINI^a^				<.001	.521^b^
Depression	1 (3%)	0	1 (3%)		
Dysthymia	5 (16%)	0	1 (3%)		
Suicidal risk	3 (10%)	0	0		
Hypomania	1 (3%)	0	1 (3%)		
Mania	2 (7%)	0	0		
Panic	4 (13%)	1 (3%)	1 (3%)		
Agora phobia	4 (13%)	0	0		
Social anxiety	5 (16%)	0	1 (3%)		
Obsessive compulsive	1 (3%)	0	0		
Post-traumatic stress	1 (3%)	0	0		
Alcohol abuse	4 (13%)	1 (3%)	1 (3%)		
Psychotic	3 (10%)	0	0		
Generalized anxiety	5 (16%)	0	3 (9%)		
Kinsey scale^a^ (Identity)				<.001	.417^b^
Attracted to the opposite ...gender	17 (56%)	29 (97%)	33 (94%)		
Attracted to both genders	5 (17%)	0	0		
Attracted to the same ...gender	8 (27%)	1 (3%)	2 (6%)		

*Note.* $\eta_{p}^{2}=\text{partial eta square}.$

^a^Chi-square has been conducted.

^b^Eta as effect size.

^c^Trans men (TM) > cis men (CM) and cis women (CW) (*P* ≤.003).

^d^Trans men > cis men (*P* = .02), the subscripts are showing the follow-up t-tests. Measurement of sexual orientation: from exclusively attracted to the opposite gender (0) to exclusively attracted to the same gender (6).

### Procedure

#### Experimental Task

**Figure 1.**
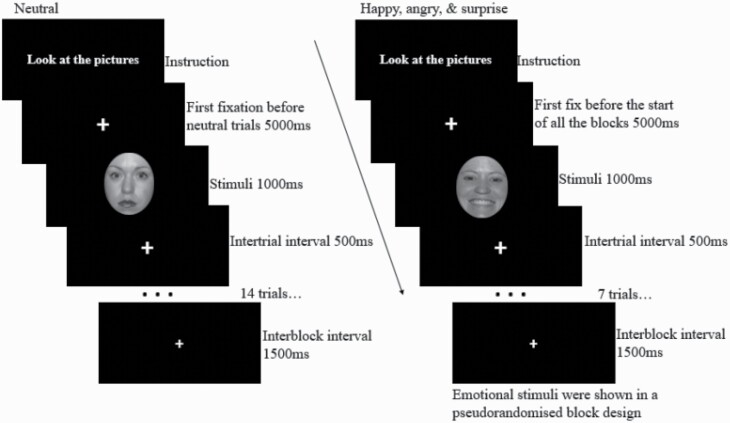
Illustration of the experimental design. Neutral (n = 14), happy (n = 7 in each block), angry (n = 7 in each block), and surprised (n = 7 in each block) emotions are counterbalanced in block design. First, the neutral emotional faces (merely 1 block) appear, and afterward all the other emotional faces are shown 3 times in blocks in the following order: first block: happy, angry, surprised; second block: angry, surprised, happy; third block: surprised, angry, happy. The fixation mark is shown each time for 500 milliseconds, whereas the emotional faces are shown for 1000 milliseconds.

Neutral, happy, angry, and surprised facial expressions of 56 identities (one-half from men, one-half from women) were selected from the NimStim standardized facial expression stimulus set (Tottenham et al., 2009). To decrease low-level visual influences, faces were (1) grey scaled, (2) presented in ovals with the hair removed, which also (3) lowered the traceability of gender (Figure 1). The stimuli were presented in a fixed counterbalanced block-design. That is, 1 block of (n = 14) neutral faces was always presented first to obtain an unbiased baseline, followed by counterbalanced blocks of 7 different faces each but with the same emotional expression (3 blocks of happy, angry, and surprised faces). Faces (visual angle 20°) were presented one at a time for 1000 milliseconds on a black background at the center of the screen with intertrial intervals (a white fixation cross) for 500 milliseconds and with an interblock interval of 1500 milliseconds (Figure 1). Stimuli were presented using E-Prime 3.0 software (Psychology Software Tools, Pittsburgh, PA, USA).

#### Testing Session

Participants who agreed to participate in the study were asked to sign their informed consent approximately 2 weeks before the actual testing. With signing the informed consent beforehand, we could ask the participants to fill out all of the questionnaires (e.g., the State-Trait Anxiety Inventory) at home at their own pace. Thus, informed consent was asked for twice: once before coming to the laboratory (online) and once during the testing session (written). Prior to the scanning session, participants underwent the Mini-International Neuropsychiatric Interview (Dutch version 5.0.0) a standardized and validated interview for investigating psychiatric disorders (Sheehan et al., 1998) taken by a clinical psychologist (M.K.). After the interview, participants were asked to fill out a pre-scanning MRI checklist to screen for standard MRI exclusion criteria (e.g., metal implants, pregnancy). In the MRI, participants viewed the stimuli via a mirror on the coil to a screen at the end of the MRI bore. Before the start of the task, to limit any head motions, participants were asked not to move their head and to look passively at the emotional faces. After the scan, participants completed a post-checklist to rule out any of the uncomfortable feelings caused by the MRI.

### Questionnaires

#### Anxiety

The State-Trait Anxiety Inventory (Spielberger et al., 1983) is a 40-item questionnaire rating both state anxiety (20 items) and trait anxiety (20 items) on a 4-point Likert scale (e.g., from “almost never” to “almost always”), where higher scores indicate greater anxiety. Current Cronbach’s α were .88 for state anxiety and .94 for trait anxiety.

#### Depression

The Beck Depression Inventory (Beck et al., 1961) (BDI) later revised (Beck et al., 1996) (BDI-II) consists of 21 items of symptoms and attitudes. Each item has 4 to 5 self-evaluative statements, which are ranked on a 4-point scale (i.e., from 0 to 3) to reflect the range of severity of depression (Beck et al., 1996). Current Cronbach’s α was .86.

#### Stress

The Perceived Stress Scale (Cohen et al., 1983) consists of 10 items about life events that were unexpected and uncontrollable. Each item is scored on a 5-point Likert scale (i.e., from 0, “never” to 4, “always”) to reflect the range of experienced stress. Cronbach’s alpha in the current sample was α = .86.

##### MRI Acquisition

Participants were scanned on a 3-Tesla Siemens Prisma Fit (Siemens, Erlangen, Germany) MRI Scanner. fMRI data during the task were acquired in 1 single run of 4:29 minutes with a field-of-view (FOV) = 208 mm^2^, slice thickness = 2 × 2 × 2 mm, TR = 1300 milliseconds, TE = 32.60 milliseconds, and flip angle = 70 degrees. A high-resolution T1-weighted MP-RAGE distortion corrected anatomical image was also acquired (duration of 6:07 minutes) in ascending order with a FOV = 256 mm^2^, slice thickness = 1 × 1 × 1 mm, TR = 2300 milliseconds, TE = 2.96 milliseconds, and flip angle = 9 degrees. A Siemens Prisma fit 64 channel head coil was used.

##### ^1^H-MRS Acquisition

Immediately following the anatomical sequence, the ^1^H-MRS sequence was run. Both water-suppressed and unsuppressed single voxel ^1^H-MRS data were collected to quantify the metabolites (Cho, N-acetyl-aspartate, glutamate, myo-inositol + glycine, and creatine). The acquisition parameters were set at TE = 75 ms, TR = 3000 ms, bandwidth = 2000 Hz, number of data points = 1024; number of averages were set to water suppressed (i.e., 128) and water unsuppressed (i.e., 8). The FOV for the amygdala (i.e., region of interest [ROI]) was set at AP = 20 mm, HF = 15 mm, and RL = 20 mm. The FOV for the ^1^H-MRS sequence (single voxel) was set to fit the box of the ROI. The voxel for the ROI was placed in the left hemisphere. With CHEmical Shift Selective pulses, water suppression data were obtained (Haase et al., 1985). A high-resolution T1-weighted MPRAGE non-distortion–corrected anatomical image was also acquired (duration of 6:07 minutes) in ascending order with a FOV = 256 mm^2^, slice thickness = 1 × 1 × 1 mm, TR = 2300 milliseconds, TE 2.96 milliseconds, and flip angle = 9 degrees. Manual and automated shimming was performed using the Siemens FASTMAP software (Gruetter, 1993) to obtain the full-width half-maximum of the water peak (i.e., 20 Hz on average). The ^1^H-MRS data, by themselves, have been published (Collet et al., 2021). Here, the data are combined with the mood and anxiety symptoms as well as neural responses to affective stimuli.

### Data Processing and Statistical Analyses

#### Task fMRI Data Processing

Raw images were first visually inspected for artifacts before preprocessing. All data were then preprocessed and analyzed with the Statistical Parametric Mapping Software (SPM12, Wellcome Department of Imaging Neuroscience, London, UK) in a Matlab Environment (The Mathworks, Natick, MA, USA). The experimental task started after the fifth scanner pulse to allow for calibration effects. Functional images were reoriented and slice time corrected, realigned to the first scan to correct within- and between-run motions, and co-registered to the T1-weighted MPRAGE anatomical image, which was normalized into a standard stereotactic space (Montreal Neurological Institute). Images were also spatially smoothed using a 6-mm FWHM Gaussian kernel.

#### ^1^H-MRS Data Processing

To be able to retrieve metabolite values from our data, the Hankel-Lanczos Singular Value Decomposition filter with model order of 25 was used to suppress the residual water peak at 4.7 ppm (Pijnappel et al., 1992; Vanhamme et al., 1998). Digital resolution of the data was increased by apodization (Lorentzian function, with factor 2 Hz) and by zero filling (with factor 2). Quantification of the data was conducted using jMRUI, and fitting of the metabolite peaks was achieved using the QUEST algorithm in jMRUI (Ratiney et al., 2004, 2005; Stefan et al., 2009). Metabolite basis sets were simulated using the nuclear magnetic resonance scope tool (Graveron-Demilly et al., 1993). The metabolite peaks were fitted at 2.01 ppm (N-acetyl-aspartate), 3.2 ppm (Cho), 2.35 and 3.74 ppm (glutamate), 3.56 ppm (myo-inositol + glycine), and 3.03 ppm (creatine). The quality of fitting was evaluating with the Cramer-Rao Lower Bound criteria. The amplitude for the Cramer-Rao Lower Bound criteria was set to <20% to eliminate poorly fitted metabolites.

#### Task fMRI Statistical Analyses

In addition to the amygdala, we also selected 3 further ROIs based on a meta-analysis (Di et al., 2017) that indicated involvement of the following regions in emotion processing: the ACC, the left middle frontal gyrus (lMFG), and the medial frontal gyrus (MFG). Anatomical ROIs (i.e., left and right amygdala) were created with the JuBrain Anatomy toolbox v. 3.0., whereas the other 3 ROIs were created with the Marsbar toolbox (Brett et al., 2002) in SPM8 with 6-mm spheres for each ROI ACC: Broadmann’s area (BA) = 32, x = 6, y = 48, z = 0 (Erk et al., 2010; Comte et al., 2016), lMFG: BA = 10, x = −36, y = 50, z = 14 (Winecoff et al., 2011; Xu et al., 2013), and MFG: BA = 32, x = −2, y = 14, z = 46 (Cohen et al., 2008; Akitsuki and Decety, 2009). Mean activation estimates (beta weights) for each condition from each participant for each ROI were then extracted and examined statistically at group level using repeated-measures ANCOVAs (rmANCOVA) in SPSS v. 26 (SPSS Inc, Chicago, IL, USA) using alpha as .05 (2-tailed). Emotion conditions (happy, angry, and surprised) were subtracted from the neutral condition to partially rule out the confounding/habituation effect (relatively higher activation level of the neutral stimuli at the beginning of the run) (Balderston et al., 2011; Plichta et al., 2014). Outliers (2*SD ± from mean) were removed prior to analyses to rule out extreme beta weights. Education level was added as a covariate to all analyses, and post-hoc follow-up tests were corrected for multiple comparisons using a step-down Bonferroni-Holm procedure (*P < *.05, 2-tailed, corrected) (Holm, 1979). Partial eta^2^ were reported as a measure of effect size where appropriate. For the ACC, no significant findings emerged and will not be further discussed.

#### ^1^H-MRS and Moderation Statistical Analyses

All of the ^1^H-MRS data were based on absolute values. Moderation analyses were conducted in SPSS v. 26, and all analyses were based on model 1 of the Process v. 3.5 extension (Hayes, 2015) for SPSS v. 26. We performed simple moderation analyses with education level as covariate. Where applicable R^2^, *t*, *b*, and Cohen’s *f*^*2*^ values are given. Only statistically significant models at *P *< .05 are reported (the case for Cho and NAA, but not for glutamate, myo-inositol, glycine, and creatine), after false-discovery-rate correction of the main effect of the model, since multiple models were run (1 for each emotion per group). Thus, all presented *P* values for the main effect are corrected values for multiple comparisons. As noted in the introduction, based on prior findings of a relationship between Cho and anxiety (Bjelland et al., 2009; Howells et al., 2015), the main analysis was geared towards this metabolite. However, other metabolites were also explored post hoc and (false-discovery rate) corrected. Findings surviving correction are reported in the supplementary Material (NAA). In addition, we also examined post hoc whether we might be able to find an association between Cho and depression (BDI-II), contrary to the null effect reported by the research group (Bjelland et al., 2009). However, similar to these researchers (Bjelland et al., 2009), we also could not find any significant findings and thus these data are not further reported. Because of the high correlation between state and trait anxiety, we only examined trait anxiety as a more stable personality trait and cumulative measure of anxiety over time (Spielberger et al., 1983). The findings for state anxiety can be found in supplementary Figures 1 and 2 but were similar to the findings in trait anxiety reported here.

## RESULTS

### Neural Responses to Emotional Stimuli

#### Amygdala

**Figure 2.**
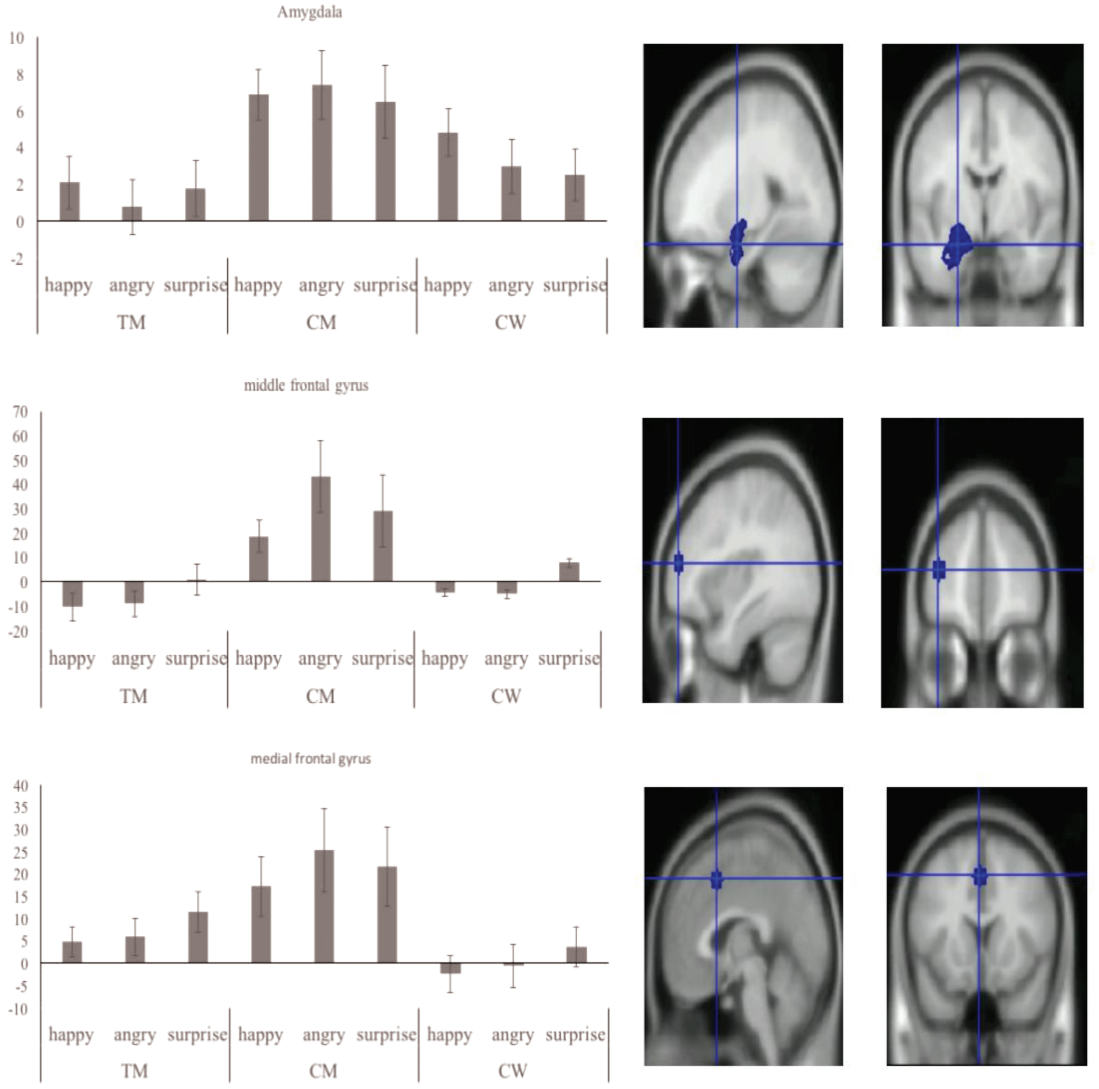
Illustration of brain activation towards happy, angry, and surprised faces (after subtraction from neutral) for each group and the amygdala (top), middle frontal gyrus (middle), and medial frontal gyrus (bottom).

The 3 (emotion: happy, angry, surprised) × 3 (group: TM, CM, CW) rmANCOVA on the left amygdala revealed a statistically significant between-group effect (F_(2,74) _=_ _3.47, *P = *.04) with a medium to large effect (${\mathrm{\eta 4}}_{p}^{2}=.08$). Post-hoc tests indicated a lower activation level of the amygdala regardless of emotion condition for TM and CW relative to CM (*P* = .032 and *P* = .038, Bonferroni-Holm corrected, respectively), indicating a sex assigned at birth pattern for TM. No significant findings emerged for the right amygdala (F_(2,72) _=_ _1.53, *P* = .22, $\eta_{p}^{2}=.04$) (Figure 2, top).

#### lMFG and MFG

The same 3 × 3 rmANCOVAs for the lMFG and MFG replicated the pattern found in the left amygdala. A main effect of group occurred in the middle frontal gyrus (F_(2,69) _=_ _6.15, *P < *.01, $\eta_{p}^{2}=.15$) indicating, post hoc, lower activation regardless of condition in TM and CW relative to CM (*P* = .001 and *P* = .014, corrected, respectively). The same pattern emerged for the MFG, with a main effect of group (F_(2,73) _=_ _4.32, *P* = .017, $\eta_{p}^{2}=.11$) and significantly lower overall activation (regardless of condition) for TM and CW relative to CM (*P* = .052 and *P* = .006, corrected, respectively) (Figure 2, middle and lower panel). No other effects were significant.

### Moderation Analyses of Anxiety, Stress, and Metabolites on Neural Responses

#### Trait Anxiety*Cho by Left Amygdala (Angry Condition)

**Figure 3.**
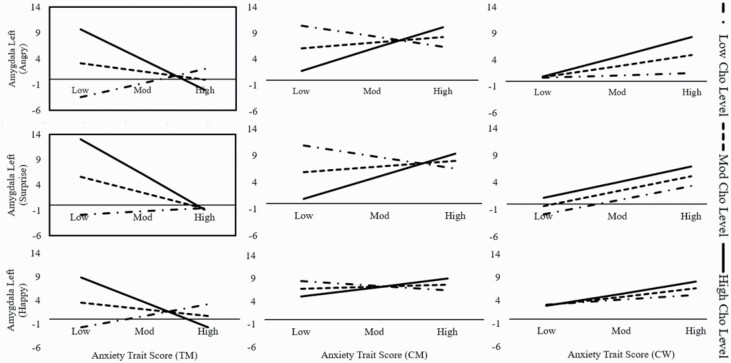
Moderation effect for trait anxiety. Figure shows the effect of Cho levels on the left amygdala for angry and surprise condition, only for TM. A unique pattern can be seen for TM relative to CM and CW. More precisely, TM with low scores on the trait anxiety and with high Cho levels will have a higher activation level of the left amygdala (angry and surprise, framed black) relative to TM with low scores on the trait anxiety and low levels of Cho. Significant models (*P < *.05) have a black frame. Cho = Choline, TM = trans men, CM = cis men, CW = cis women.

The moderation analysis with trait anxiety as independent variable, choline levels as moderator effect, and left amygdala activation to angry faces revealed a statistically significant effect of our model (*F*_(4, 17)_ = 3.37, *P* = .03, *R*^2^ = .44, *f*^2^ = .79) and, crucially, an interaction effect of trait anxiety*Cho on the left amygdala (*b* = −4.96, *t*_(17)_ = −3.22, *P < *.01). Importantly, this effect was present only in TM but not in cisgender comparisons (Figure 3; supplementary Table 1). This statistically significant moderating model indicated that TM low in trait anxiety but with high Cho levels had a higher activation level of the left amygdala to angry faces relative to TM low in trait anxiety and low levels of Cho. No other effects were found.

#### Trait Anxiety*Cho by Left Amygdala (Surprised Condition)

Similar to the effect for angry faces, a similar main effect of the model—again for TM only—emerged for the surprised condition (*F*_(4, 17)_ = 6.05, *P* = .03, *R*^2^ = .59, *f*^2^ = 1.44). Likewise, the interaction effect of trait anxiety*Cho on the left amygdala (surprise) was also significant (*b* = −4.19, *t*_(17)_ = −3.24, *P < *.01). In addition, main effects of trait anxiety (*b* = −.3, *t*_(17)_ = −2.58, *P* = .02) and Cho (*b = *42.08, *t*_(17)_ = 2.63, *P* = .02) also emerged (Figure 3; supplementary Table 1). The pattern found for angry faces was thus replicated for surprise faces, that is, TM low in trait anxiety and high Cho levels had a higher neural activation than TM low on both anxiety and Cho. Importantly, this pattern was only present for negative (angry) and ambiguous (surprised) emotions (both *P < *.01) but did not emerge for the happy faces (*P* > .05).

#### Perceived Stress*Cho by Left Amygdala (Surprised Condition)

**Figure 4.**
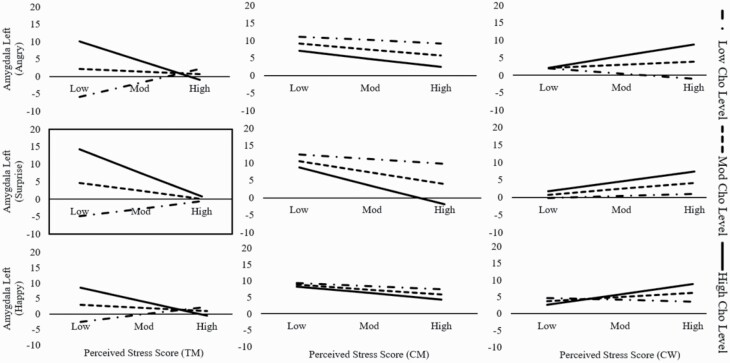
Moderation effect for perceived stress. Merely the moderation effect of Cho was significant on the left amygdala (surprise) in the TM group. Significant model (*P < *.05) have a black frame. Cho = Choline, TM = trans men.

This moderation model was statistically significant for TM (but not CM or CW) with *F*_(4,17)_ = 3.04, *P* = .0462, *R*^2^ = .42, *f*^2^ = .72 for the model and with *b* = −7.61, *t*_(17)_ = −2.73, *P* = .03 for the interaction effect. The model showed the same pattern/effect as for trait anxiety; here low stress scores and high Cho levels had a higher neural activation than TM low on both stress and Cho. Moreover, this interaction of perceived stress*Cho on the activation level of the left amygdala was only significant during the surprised condition. The main effect of Cho (*b* = 58.71, *t*_(17) _=_ _2.78, *P* = .01) was also significant (Figure 4; supplementary Table 1). No other effects of group or metabolites were significant.

## Discussion

This study examined the neural correlates and metabolite concentrations in TM experiencing minority stress relative to a cis comparison group. Based on previous findings, the main 2 hypotheses were that unlike the cis comparison group, (1) TM would show a larger neural activation in the amygdala to negative stimuli, and (2) this effect would be modulated by Cho concentrations. As for the first hypothesis, TM did not show a larger neural activation in the amygdala to negative stimuli but a pattern similar to CW. With regards to the second hypothesis, as predicted, Cho modulated neural responses to negative and ambiguous but not to positive emotions, and only in TM but not cis people.

### Moderating Effect of Cho on Amygdala Activation Levels

In accordance with prior work (Howells et al., 2015), we found an interesting moderation of Cho on anxiety, stress, and neural activation. Specifically, high concentrations of Cho alongside low scores of trait anxiety showed an increased neural activation in the left amygdala for negative and surprised faces but not happy faces. Importantly, this effect only emerged in TM but not cis people, and although limited studies (Mukai et al., 2014; Su et al., 2018) have reported a modest involvement of myo-inositol on anxiety, this was not the case in our study sample. A similar effect was present for perceived stress albeit only in the surprise condition. This novel finding highlights the interaction among anxiety, stress, and Cho and their influence on the amygdala signal during processing of negative emotions. Consistent with the idea for an overgeneralization (Lissek et al., 2014) and the hypothesis for increased vigilance toward the environment to ward off potential negative experiences (e.g., discrimination, violence) in sexual minorities (Meyer 2003), the pattern found for negative faces extended to emotionally ambiguous faces (surprise). It thus suggests that trans people may indeed be processing emotionally ambiguous faces as potential threatening or dangerous, showcasing a putative neural marker of continued minority stress.

One consequence of such continued minority stress is, as noted in the introduction, that sexual minority groups suffer from higher rates of anxiety (Heylens et al., 2014; Cohen et al., 2016) and exhibit higher perceived stress (Meyer 2003). In addition to further confirming these findings in a group of TM prior to commencing gender-affirming treatment, the present results add to prior work in the neural domain. The present associations of Cho with anxiety were nicely consistent with prior findings (Bjelland et al., 2009) that also showed associations with plasma Cho concentration and anxiety but no association of Cho with depression. We are thus replicating both of their findings. However, different from these same researchers (Bjelland et al., 2009), our analyses added a level of complexity further examining how the relationship between Cho and anxiety would influence neural responses in affective neurocircuitry. Presently, the literature on how, mechanistically, Cho may influence the blood-oxygen-level-dependent response is scarce.

Two studies from the same laboratory might shed some light on this issue. In a series of experiments in humans and rats to assess goal-directed behavior, a research group (Howe et al., 2013) showed that prefrontal brain activity seemed to have been directly influenced by Cho emissions. In a second follow-up study (Berry et al., 2015), genetic polymorphisms implicated in cholinergic function in a human sample suggested reduced prefrontal activation during the goal-directed behavior task in the low cholinergic polymorphism group. Of note, both studies had to rely on various indirect methods to assess the relationship between neural activation and Cho activity; in the first study by examining and relating Cho activity and oxygen consumption through microelectrodes in rodents and in the second study by relying on a genetic polymorphism in a human sample. The present study and findings add value to this scarce body of work because it shows how ^1^H-MRS combined with fMRI can also yield important insight into the respective relationship. As the 2 cited studies (Howe et al., 2013; Berry et al., 2015) above show, examining how exactly Cho modulates cognitive-affective function is difficult, and in humans, it has been measured only indirectly. Given the broad involvement of Cho on brain function, further inquiry is needed regarding the underlying mechanisms and routes of Cho on cognitive-affective processing.

### Neuroimaging Findings

Indeed, the modulation and influence of Cho and anxiety on neural responding might explain why we did not find variability in neural activation to emotion per se or a stronger neural effect for TM as hypothesized. Instead, in the fMRI data, neural responses were similar across all tested brain regions (left amygdala, lMFG, and MFG), with no findings for the ACC. In addition, TM indicated a sex assigned at birth pattern, meaning that both TM and CW did not differ from each other but had lower overall activation relative to CM. One possibility is that any main effects were masked by the moderation with metabolites. Although speculative at this point, while TM showed significant interactions among Cho, anxiety, and amygdala response to negative stimuli, CW tended to show a linear effect (not significant, cf. Figure 3), indicating slightly higher neural response regardless of Cho level with more anxiety. Thus, the underlying interaction in TM may have masked any potential main effects that would/could differentiate them from CW. Alternatively, a general absence of neural differences among the emotion conditions across all groups may be consistent with an earlier finding in a relatively large sample of healthy and maltreated adults that could also not establish any such neural differences (van Harmelen 2013). Moreover, a sex-assigned-at-birth pattern in hormone naïve TM would also be consistent with some prior functional MRI studies (Gizewski et al., 2009; Burke et al., 2014) and structural MRI studies (Savic and Arver 2011; Zubiaurre-Elorza et al., 2013; Sorouri Khorashad et al., 2020). Whereas no clear gender differences emerged in the emotion study by researchers (Beking et al., 2020) in trans youth, another research group (Fisher et al., 2020) revealed a pattern consistent with gender identity in TM in a face perception task. However, their study (Fisher et al., 2020) focused on gender perception and structures of the parietal lobe rather than emotion processing. In conclusion, the neural pattern in TM resembled that of their sex assigned at birth, with no differences among emotion conditions. However, the precise reason for this effect remains to be further investigated and validated.

### Limitations

A first limitation is the cross-sectional nature of the study and thus prohibits any causal inferences. A second methodological limitation is the spectroscopic voxel placement. Because of the small structure of the amygdala and surrounding tissue (cerebrospinal fluid), we had to extend the box to the anterior hippocampus, which yielded much better signal-to-noise during piloting than scanning the amygdala alone. Although this much improved data quality, it restricts anatomical inference. Therefore, the metabolite findings should be interpreted with caution and as being a sample of the combined amygdala and anterior hippocampus metabolite levels. Another methodological limitation was that due to time constraints and other sequences acquired during the session, we were only able to collect ^1^H-MRS data on the left (but not right) amygdala. Therefore, future work would need to examine laterality issues when examining metabolite effects on neural circuitry. Another limitation that deserves attention is the role of Cho in inflammation and where inflammation has been linked to depression and anxiety (Peirce and Alviña 2019). The role of inflammation, though highly interesting, was not examined in the present paper (e.g., by examining metabolites of the Cho phospholipids pathway). Speculatively, 2 reasons why inflammation may not have played a strong role are (1) no effects of depression were apparent despite links between depression and inflammation (Beurel et al., 2020); and (2) no effects of myo-inositol, which has also been linked to inflammation (De Paepe et al., 2020), have emerged. Nonetheless, given the methodological limitations of the current design, we encourage future researchers to take this point into account. Lastly, both fMRI and ^1^H-MRS sequences were acquired separately; thus, the concentration of the metabolites reflects an unconstrained resting state rather than reflecting metabolite variation during the task. The fact that Cho levels nonetheless significantly and selectively modulated the findings, however, speaks to the relevance it may play in neural responding to emotional faces.

## Conclusions

In conclusion, in this combined fMRI-^1^H-MRS study, we demonstrated a moderating effect of Cho on the effect of anxiety and perceived stress and the activation level of the left amygdala that was present in TM but not cisgender comparisons. Moreover, moderation by Cho occurred only during angry and surprised conditions, suggesting an effect of minority stress in TM. Contrary to expectations, TM did not show larger neural activations in the amygdala to negative stimuli but indicated a similar pattern to CW and different from CM. This study provides new data to our understanding of how brain metabolites may influence neural responding to emotions, particularly in minority stress groups.

## Supplementary Material

pyab090_suppl_Supplementary_Figure_S1

pyab090_suppl_Supplementary_Figure_S2

pyab090_suppl_Supplementary_Table_S1

## Interest Statement

None of the authors has a conflict of interest to declare. The funding sources had no influence on the research design, data collection, analyses and results, or interpretation.
