# Supplementary figures and images for: Minority Stress and the Effects on Emotion Processing in Transgender Men and Cisgender People: A Study Combining fMRI and 1H-MRS
Source: Int J Neuropsychopharmacol. 2021 Dec 8;25(5):350–60. doi: 10.1093/ijnp/pyab090 (PMC9154245; doi:10.1093/ijnp/pyab090)

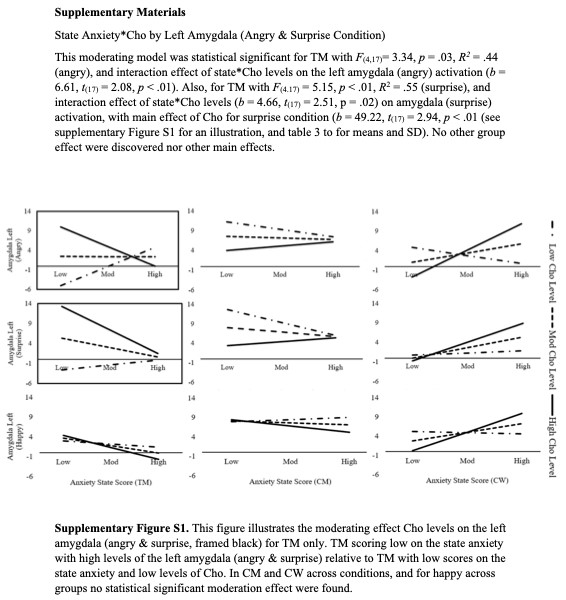

Supplement: pyab090_suppl_Supplementary_Figure_S1 [file pyab090_suppl_supplementary_figure_s1.jpeg]

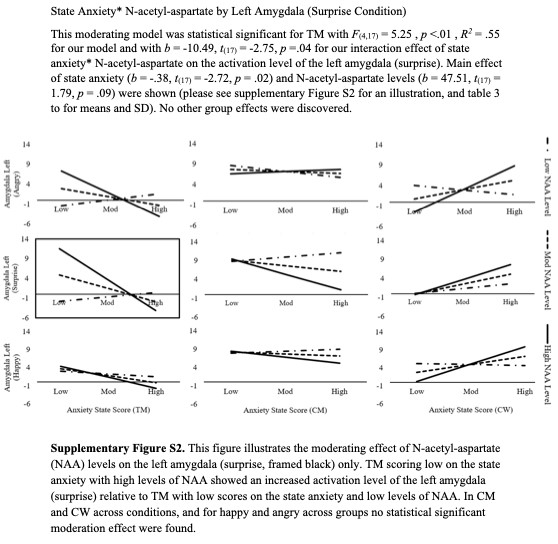

Supplement: pyab090_suppl_Supplementary_Figure_S2 [file pyab090_suppl_supplementary_figure_s2.jpeg]

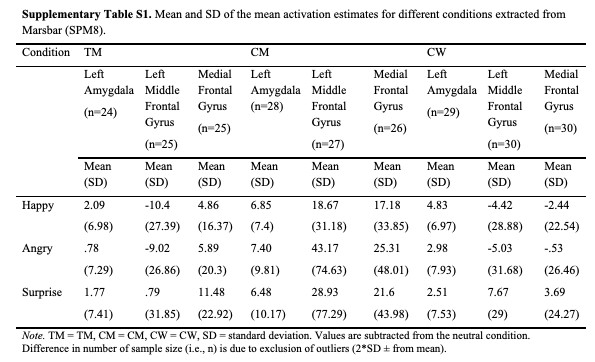

Supplement: pyab090_suppl_Supplementary_Table_S1 [file pyab090_suppl_supplementary_table_s1.jpeg]
